# A retrospective on lithium-ion batteries

**DOI:** 10.1038/s41467-020-16259-9

**Published:** 2020-05-19

**Authors:** Jing Xie, Yi-Chun Lu

**Affiliations:** Electrochemical Energy and Interfaces Laboratory, Department of Mechanical and Automation Engineering, The Chinese University of Hong Kong, Shatin, 999077 Hong Kong SAR, China

**Keywords:** Batteries, Batteries, Batteries

## Abstract

The 2019 Nobel Prize in Chemistry has been awarded to John B. Goodenough, M. Stanley Whittingham and Akira Yoshino for their contributions in the development of lithium-ion batteries, a technology that has revolutionized our way of life. Here we look back at the milestone discoveries that have shaped the modern lithium-ion batteries for inspirational insights to guide future breakthroughs.

The rechargeable lithium-ion batteries have transformed portable electronics and are the technology of choice for electric vehicles. They also have a key role to play in enabling deeper penetration of intermittent renewable energy sources in power systems for a more sustainable future. A modern lithium-ion battery consists of two electrodes, typically lithium cobalt oxide (LiCoO_2_) cathode and graphite (C_6_) anode, separated by a porous separator immersed in a non-aqueous liquid electrolyte using LiPF_6_ in a mixture of ethylene carbonate (EC) and at least one linear carbonate selected from dimethyl carbonate (DMC), diethyl carbonate (DEC), ethyl methyl carbonate (EMC) and many additives. During charging, Li-ions move from the LiCoO_2_ lattice structure to the anode side to form lithiated graphite (LiC_6_). During discharging, these ions move back to the CoO_2_ host framework, while electrons are released to the external circuit. It is this shuttling process or what is called rocking-chair chemistry that has revolutionized our modern life.

## Materials discoveries

### Anode

**Fig. 1:**
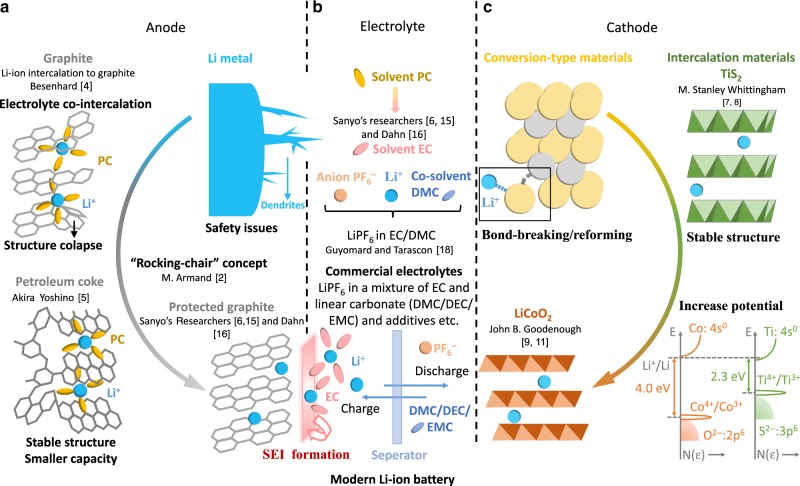
Milestone discoveries that shaped the modern lithium-ion batteries. The development of (**a**) anode materials including lithium metal, petroleum coke and graphite, (**b**) electrolytes with the solvent propylene carbonate (PC), a mixture of ethylene carbonate (EC) and at least one linear carbonate selected from dimethyl carbonate (DMC), diethyl carbonate (DEC), ethyl methyl carbonate (EMC) and many additives, (**c**) cathode materials including conversion-type materials, intercalation materials titanium disulfide (TiS_2_) and lithium cobalt oxide (LiCoO_2_).

Lithium metal is the lightest metal and possesses a high specific capacity (3.86 Ah g^−^^1^) and an extremely low electrode potential (−3.04 V vs. standard hydrogen electrode), rendering it an ideal anode material for high-voltage and high-energy batteries. However, the electrochemical potential of Li^+^/Li lies above the lowest unoccupied molecular orbital (LUMO) of practically known non-aqueous electrolytes, leading to continuous electrolyte reduction unless a passivating solid electrolyte interface (SEI) is formed^1^. The SEI is susceptible to damage and repairs nonuniformly on the surface of lithium metal owing to the large volume change and high reactivity of lithium metal, leading to dendrite growth, which could cause cell to short-circuit and catch fire (Fig. 1a).

To avoid safety issues of lithium metal, Armand suggested to construct Li-ion batteries using two different intercalation hosts^2,3^. The first Li-ion intercalation based graphite electrode was reported by Besenhard showing that graphite can intercalate several alkali-metal ions including Li-ions^4^. Graphite intercalates Li-ions based on a layered structure with half-filled p_z_ orbitals perpendicular to the planes that can interact with the Li 2s orbitals to limit volume expansion and dendrite growth. However, the specific capacity of graphite (LiC_6_, 0.372 Ah g^–1^)^1^ is much smaller than that of lithium metal. It was until a total recall of lithium metal batteries by Moli Energy after several fire accidents that intercalation materials such as graphite were increasingly viewed as a viable anode in the race to replace lithium metal for better safety. At that time, co-intercalation of electrolyte (propylene carbonate PC) led to exfoliation and collapse of the graphite (Fig. 1a), posing a challenge for its application in a battery cell.

In 1985, Akira Yoshino^5^ at Asahi Kasei Corporation discovered that petroleum coke, a less graphitized carbon from the residual of petroleum fractionation, can reversibly intercalate Li-ions at a low potential of ~0.5 V relative to Li^+^ /Li without structural destruction. Its structural stability originates from the amorphous carbon regions in petroleum coke serving as covalent joints to pin the layers together^6^ (Fig. 1a). Although the amorphous nature of petroleum coke limits capacity compared to graphite (~Li_0.5_C_6_, 0.186 Ah g^–1^)^6^, it became the first commercial intercalation anode for Li-ion batteries owing to its cycling stability.

### Cathode

To cater to the high capacity of lithium metal, conversion-type cathodes including metal fluorides, sulfides or oxides (Fig. 1c) were considered at first. During battery operation, these materials react to form phases with different structures and new compositions^6^. Consequently, conversion electrodes do not allow for many cycles since bond breaking and reforming occur during each cycle.

Knowing the limitation of conversion reactions, scientists turned to new lithium ion storage mechanisms that involve no structural collapse during cycling. Metal chalcogenides (MX_2_) with a layered structure and available space to store Li-ion guests received attention from Whittingham and co-workers at Exxon^7^ who showed that titanium disulfide (TiS_2_) can chemically intercalate Li-ions over its entire stoichiometric range with minimized lattice expansion. In 1973 and 1974 Whittingham applied TiS_2_ as the cathode for batteries (Fig. 1c) and subsequently demonstrated a 2.5 V cell in 1976^8^.

Apparently, the low voltage of the TiS_2_//Li battery indicates that its energy density is limited. Aiming to find new cathode materials that intercalate Li-ions at higher potentials, Goodenough turned to the oxide equivalents of metal chalcogenides (MX_2_, where X = O). He noted that the top of the S-3p^6^ bands is higher in energy than that of the O-2p^6^ bands, which renders higher intercalation potentials for metal oxides than metal sulfides^9^ (Fig. 1c). The higher energy of the S-3p^6^ bands in metal sulfides is attributed to a smaller electrostatic Madelung energy (larger sulfide ion), and a greater energy required to transfer an electron from the cation (M^n+^) to S^–^/S^2–^ at infinite separation^9^.

This basic understanding led to the discovery of three classes of oxide cathodes by Goodenough and co-workers^10^. In 1979 and 1980, Goodenough reported a lithium cobalt oxide (LiCoO_2_)^11^ which can reversibly intake and release Li-ions at potentials higher than 4.0 V vs. Li^+^ /Li and enabled a 4.0 V rechargeable battery when coupled with lithium metal anode. However, cobalt has limited abundance, forming a cost barrier to its application. The spinel LiMn_2_O_4_^12^ with tetrahedral-site lithium ions offers a redox potential of ~4.0 V vs. Li^+^/Li with a reduced cost. However, it is limited by the degradation issues owing to the Mn dissolution in the presence of H^+^ ions (ppm level) in the electrolyte. The polyanion oxide Li_x_Fe_2_(*X*O_4_)_3_ (*X* = S, Mo, W, etc.)^13,14^ offers higher cell voltage compared to simple oxides such as Fe_2_O_3_/Fe_3_O_4._ The covalent *X*-O bond in the polyanion oxide weakens the covalency of Fe-O bond through inductive effect, leading to a lowered redox energy of the Fe^2+/3+^ couple and thus an increase in the redox potential (for example, from <2.5 V in Fe_2_O_3_ to 3.6 V in Li_x_Fe_2_(SO_4_)_3_). Polyanion oxide provides advantages of a reduction in cost with abundant transition metals like Fe, and improved thermal stability and safety owing to the tight covalent bonding of oxygen. However, it suffers from a poor electronic conductivity and lower densities. Among the three classes of oxides, layered oxides with high gravimetric and volumetric energy densities remain as the favorite cathodes so far^10^, and the LiCoO_2_ electrode is now the dominant cathode material that powers most personal electronic devices.

### Electrolyte

The working window of an electrolyte is determined by its LUMO and highest occupied molecular orbital (HOMO), which should be higher than the electrochemical potential of anode (*μ*_a_) and lower than the electrochemical potential of cathode (*μ*_c_), respectively (LUMO > *μ*_a_, HOMO < *μ*_c_). Alternatively, a stable passivating SEI layer should be created on the anode or cathode in the case of LUMO < *μ*_a_ or HOMO > *μ*_c_, respectively^1^.

Creating a stable SEI by tailoring electrolyte composition enabled the practical application of graphite anode. Initially, PC was preferred over ethylene carbonate (EC) owing to its lower melting temperature (−48.8 ^o^C) compared to EC (36.4 ^o^C)^6^. However, PC was reported to cause structural damage of graphite, leading to poor cycle life. Sanyo’s researchers^6,15^ claimed successful electrochemical lithiation of graphite in EC-based electrolytes, and Dahn^16^ reported that EC can suppress the graphite exfoliation due to the formation of sacrificial SEI, paving the way for the development of graphite anode for Li-ion batteries (Fig. 1b). Since then, EC became an indispensable solvent for Li-ion batteries. Potential mechanisms underlying the “EC − PC Disparity”^17^ are attributed to the differences between their reduction products. The curving chain structure of lithium propylene decarbonate (PC’s reductive product) is prone to create loose deposits with poor cohesion on the electrode surface. EC tends to form a graphite intercalation compound with higher anion (e.g. PF_6_^–^) population in Li^+^ solvation shell than PC, leading to higher F-containing SEI product, whose energy gap between LUMO and HOMO is large enough to insulate the electron tunneling from anode, rendering effective passivation of electrolyte decomposition.

## Full-cell Li-ion batteries

Asahi Kasei Corporation assembled a full rechargeable battery combining the petroleum coke anode with Goodenough’s LiCoO_2_ cathode, which was later commercialized by Sony in 1990 (~80 Wh kg^–1^, 200 Wh L^–1^) (Fig. 1). The finding of Sanyo’s researchers^6,15^ and Dahn’s work^16^ with EC as co-solvent paved the way for the development of Li-ion batteries with a graphite anode and increased the voltage and energy density to 4.2 V and 400 Wh L^–1^ respectively. In 1993, Guyomard and Tarascon^18^ reported a new electrolyte formulation, LiPF_6_ in EC/DMC, for its improved oxidation stability (Fig. 1b). This electrolyte remains one of the popular electrolytes until today, affording LiCoO_2_-based Li-ion batteries three times higher energy density (250 Wh kg^–1^, 600 Wh L^–1^) than that of the first-generation devices by Sony^3^.

## Summary

None of these landmark discoveries appeared out of thin air. A success at a later stage is usually built upon previous knowledge and progress. The phenomenon that TiS_2_ can host Li-ions was observed by Walter Rüdorff as early as in 1965^19^, but this layered disulfide was not applied as a cathode in lithium batteries until 1973 by Whittingham^7^. The demise of the TiS_2_//Li effort then inspired Goodenough to look at layered oxides for the design of cathodes with a higher electrode potential. Similarly, graphite was reported to intercalate Li-ions in the 1970s^4^, but it was not practical until the work of Sanyo’s researchers^6,15^ and Dahn^16^ introducing co-solvent EC. The move was based on the in-depth understanding of the degradation mechanisms. Reflections on how these breakthroughs were born teach the critical importance of mechanistic understanding and cross-disciplinary studies.

Using lithium metal anode was the natural choice in the community during 1970s to 1980s for its high-energy advantage despite its high reactivity and dendrites issues. Calling out to use different intercalation materials for cathode and anode by Armand^2,3^ or replacing lithium metal by petroleum coke by Yoshino^5^, while seemingly divergent to the pursuit of high energy batteries, proved necessary steps toward the commercialization of rechargeable Li-ion batteries. These developments encourage us to be open-minded and dare to challenge the existing wisdom for disruptive innovation in battery designs.

The impact of lithium-ion batteries is poised to go beyond portable electronics to domains that matter to the sustainability of the society. To meet the ever-growing demand for electrified transportation and large-scale energy storage solutions, continued materials discoveries and game-changing chemistry hold the key to unleashing the full potential of lithium-ion batteries toward seriously enhanced cost efficiency, power and energy densities, and safety.
